# Anti-cancer properties of gastropodan hemocyanins in murine model of colon carcinoma

**DOI:** 10.1186/s12865-014-0034-3

**Published:** 2014-08-29

**Authors:** Vera Gesheva, Stela Chausheva, Nikolina Mihaylova, Iliyan Manoylov, Lyuba Doumanova, Krassimira Idakieva, Andrey Tchorbanov

**Affiliations:** The Stephan Angeloff Institute of Microbiology, Bulgarian Academy of Sciences, Acad. G. Bonchev Str. 26, 1113 Sofia, Bulgaria; Institute of Organic Chemistry, Bulgarian Academy of Sciences, Acad. G. Bonchev Str., bl. 9, 1113 Sofia, Bulgaria

**Keywords:** C-26 carcinoma, Murine cancer model, *Rapana thomasiana*, Helix pomatia, Hemocyanins, Anti-cancer activity

## Abstract

**Background:**

Various immunotherapeutic approaches have been used for the treatment of cancer. A number of natural compounds are designed to repair, stimulate, or enhance the immune system response. Among them are the hemocyanins (Hcs) - extracellular copper proteins isolated from different arthropod and mollusc species. Hcs are oxygen transporter molecules and normally are freely dissolved in the hemolymph of these animals. Hemocyanins are very promising class of anti-cancer therapeutics due to their immunogenic properties and the absence of toxicity or side effects. KLH (*Megathura crenulata* hemocyanin) is the most studied molecule of this group setting a standard for natural carrier protein for small molecules and has been used in anti-tumor clinical trials.

**Results:**

The Hcs isolated from marine snail *Rapana thomasiana* (RtH) and the terrestrial snail *Helix pomatia* (HpH) express strong *in vivo* anti-cancer and anti-proliferative effects in the developed by us murine model of colon carcinoma. The immunization with RtH and HpH prolonged the survival of treated animals, improve humoral anti-cancer response and moderate the manifestation of C-26 carcinoma symptoms as tumor growth, splenomegaly and lung metastasis appearance.

**Conclusion:**

Hemocyanins are used so far for therapy of superficial bladder cancer and murine melanoma models. Our findings demonstrate a potential anti-cancer effect of hemocyanins on a murine model of colon carcinoma suggesting their use for immunotherapy of different types of cancer.

## Background

Cancer is one of the major reasons for human death in the last decade following cardiovascular diseases, infectious diseases and ischemic heart diseases. Current therapies of cancer include chemotherapy and radiotherapy although they both have severe side effects. Each type of cancer requires specific treatment, which explains the need for development of highly specific targeted anti-cancer agents [1].

The BCG (Bacillus Calmette-Guérin) vaccine for tuberculosis that contains attenuated *Mycobacterium bovis* is one of the most extensively used immunotherapeutics based on its strong non-specific immunostimulatory properties [2,3]. BCG is widely administrated in cases of colorectal, lung cancers and melanoma.

In the last years a number of therapeutic antibodies have been approved for clinical treatment in cases of breast cancer (Trastuzumab), non-Hodgkin (Rituximab) and Hodgkin lymphoma (Brentuximab vedotin), colorectal cancer (Panitumumab, Cetuximab), chronic lymphocyte leukemia (Alemtuzumab), and acute myelogenous leukemia (Gemtuzumab ozogamicin) [4]. Immunotherapy with cytokines is another option for cancer treatment. IL-2 in metastatic melanoma and renal cell carcinoma and IFN-alpha in Stage III melanoma have been permitted for cancer therapy [5].

Different cancer vaccines have been developed for prevention and treatment of malignant diseases but only Sipuleucel-T is approved for therapy of advanced prostate cancer in case hormonal treatment is ineffective [6]. Many of the developed vaccines include cancer carbohydrate antigens chemically conjugated to carrier protein with or without other adjuvant [7–9].

Today natural products and their compounds derived from fungi, plants or microbes are of great interest for an anti-cancer research. This huge variety of chemical structures provides different mechanisms of action and specific effects used for anti-tumor therapy.

Marine compounds are known to have a serious potential as anti-cancer drugs. Most of them are small molecules, inhibitors of key enzymes for carcinogenesis like matrix metalloproteinazes (MMPs), HIFs, topoisomerase, protein kinase C (PKC) or transcripion factors like NFkB [10]. Special attention is payed on marine-derived anti-angiogenesis products, which suppress and prevent the succesful formation of vascular system, supporting tumor growth and invasion. The majority of these substances act via inhibition of enzymes or factors, crucial for the process of angiogenesis [11].

Another group of potential anti-cancer agents are the hemocyanins (Hcs) - oligomeric copper-containing glycoproteins that function as oxygen carriers in the hemolymph of several molluscs and arthropods. Molluscan Hcs have been studied intensively for many years with respect to their structure and function [12]. The huge molecular size (4 to 8 MDa) of molluscan Hcs, their xenogenic character and carbohydrate content have been implicated in inducing strong immune response in mammals, which has led to the biomedical and therapeutic application of these proteins. Thus, keyhole limpet hemocyanin (KLH) isolated from marine gastropod *Megathura crenulata* is a well-established immune stimulant, hapten carrier and tumor vaccine carrier [13,14].

It is known that Hcs stimulate the immune system non- specifically by interacting with macrophages, polymorphonuclears, CD4+ and CD8+ cells giving rise to potent humoral and cellular immune response. One possibility for their anti-cancer effect is that they generally enhance statement of immune system that helps the immune cells to recognize self from non-self and to eliminate non-self cancerous cells. KLH and CCH (*Concholepas concholepas* hemocyanin) are intensively studied for their anti-cancer properties in superficial bladder cancer (SBC) and in melanoma models [15,16].

Recently, we have demonstrated that the Hc isolated from marine gastropod *Rapana thomasiana* (RtH) possesses a high immunogenicity as a single model antigen and can be used as a protein carrier for viral peptides from influenza hemagglutinin [17]. Our results have shown that RtH is acceptable as a potential bio-adjuvant for subunit bacterial and viral vaccines [18]. Compared to the numerous studies on the structural organization and protein stability of the Hc isolated from garden snail *Helix pomatia* (HpH), its immunological properties have been less investigated [19]. These results point on the potential properties of RtH and HpH as anti-cancer agents.

C-26 murine model of colorectal carcinoma is classical tumor model, developed in 1975 and determinated as an undifferentiated Grade IV carcinoma [20]. C-26 cells display high tumorigenicity and low tendency to metastasize mainly in the lungs. Inoculated in syngeneic Balb/c mice C-26 cells cause high mortality. This model is used more than 35 years for studying the process of carcinogenesis and anti-cancer therapy.

The aim of the present work was to develop an experimental murine model of colon carcinoma and to investigate the anti-tumor activity of RtH and HpH.

## Results

### Hemocyanins analysis

**Figure 1:**
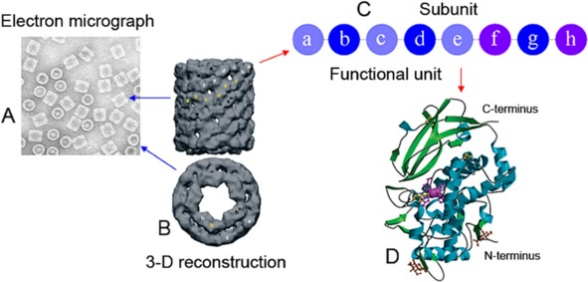
**Molecular structure of gastropod Hcs. (A)** TEM of negatively stained RtH molecules; **(B)** 3-D reconstruction of the hollow cylindrical RtH didecamer; side view and top view [21]; **(C)** scheme of the subunit containing eight different functional units; **(D)** X-ray structure of functional unit RtH2-e [22].

Figure 1 presents the synopsis of our results from the structural studies of the Hc isolated from marine snail *R. thomasiana*. The electron micrograph shows typical for gastropodan Hcs didecamers (Figure 1A). The quaternary structure of molluscan Hcs is a hollow cylindrical molecule, about 35 nm in diameter, with an internal collar complex. 3-D reconstruction of the RtH molecule is shown on Figure 1B [21]. Molecules of molluscan Hcs are structured as decamers (in cephalopods) or didecamers (in gastropods) of subunits with molecular mass of 350–450 kDa. The polypeptide chains of subunits contain seven or eight globular functional units (FUs), depending on the species. FUs are generally termed (a - h), starting from the N-terminus of the respective subunit (Figure 1C). The FUs have a binuclear copper active site, capable of binding reversibly one oxygen molecule. The active site consists of two closely spaced copper ions (Cu_A_ and Cu_B_) each coordinated by three histidine ligands (type 3 copper site). Figure 1D shows the crystal structure of a FU RtH2-e [22]. The different type of FUs share sequence identities of around 45%. Being similar in primary, tertiary and quaternary structure, molluscan Hcs differ in their carbohydrate content (between 2% and 9% (w/w)) and monosaccharide composition [23]. Most probably the specific carbohydrate content and composition of molluscan Hcs are the structural basis for the difference in their antigenicity.

### Hemocyanins increase tumor cells apoptosis *in vitro*

To study the apoptotic effect of gastropodan Hcs on tumor cells we tested their ability to influence directly the C-26 cells by apoptosis assay. C-26 carcinoma cells were incubated with different amounts of HpH and RtH (ranging from 0.1 μg/ml to 100 μg/ml). The cells were further washed and the surface expression of phosphatidylserine was studied at the 24 h, 48 h and 72 h of incubation. The levels of induced early and late apoptosis as well as necrosis were analyzed by Annexin-V/Propidium iodide staining and flow cytometry.

A significant increase in percentage of early apoptosis and a slight increase of late apoptosis were observed after 48 hours of incubation with all tested amounts of Hcs. In contrast, no obvious effect of the treatment was found on the 24 h of incubation (data not shown).

**Figure 2:**
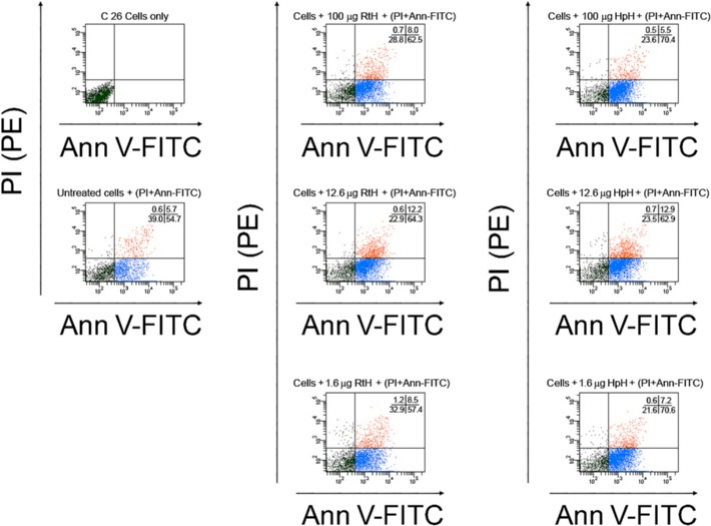
**The gastropod Hcs RtH and HpH induce apoptosis of C-26 carcinoma cell line** ***in vitro*** **.** The C-26 cells were incubated in the presence of different concentrations of HpH and RtH (100 μg/ml, 12.6 μg/ml and 1.6 μg/ml) for 72 hours at 37°С/5% CO_2_. At the end of the cultivation the cells were double-stained with Annexin V-FITC /Propidium iodide and were analyzed by flow cytometry. Percentage of stained cells is shown in each quadrant. The panels represent the results from 3 independent experiments.

High level of early apoptosis and considerable enhancement in late apoptosis was observed on the 72 h of incubation (Figure 2). Dose-dependant quantitative corelation between induced apoptosis and Hcs added to the cells was not found at all tested time points.

### Tumor model development

For selection of proper C-26 murine colon carcinoma model 4 groups of mice challenged with different cell numbers were monitored (for details see Methods section). Formation of solid tumors in all mice in the group as well as 0% survival within 3 months after cells challenge was the leading parameters for successful model establishment. In the group inoculated with 5×10^5^ cells palpable solid tumors were observed 2 weeks after C-26 challenge followed by 0% survival 3 months after cell challenge, which is acceptable for the model requested. In the other groups mice developed tumors and died too early or survived for longer period, so they weren’t appropriate for further studies.

### Phenotyping of spleen and tumor suspensions

**Figure 3:**
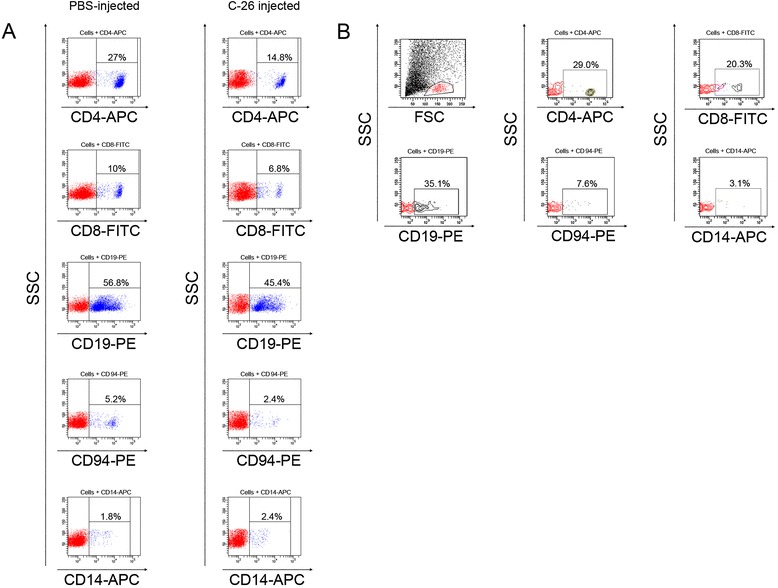
**FACS analysis of spleen and tumor suspensions.** Spleens **(A)** and solid tumors **(B)** from representative number of animals (n = 4 to 6) were excised and the cell suspensions were incubated with one of the following anti-mouse antibodies - anti-CD4-APC, anti-CD8-FITC, anti-CD19-PE, anti-CD94-PE or anti-CD14-APC. Ten thousands cells were analyzed from each sample by flow cytometry. Data are representative of at least 5 experiments.

We have performed an immune system modulation analysis during tumor development when the tumor model was established to demonstrate the immune reaction to the tumor growth. Spleens from mice challenged with 5×10^5^ C-26 carcinoma cells or PBS treated only were isolated and analyzed by flow cytometry. We observed a decrease of CD4+, CD8+ (T cells), CD19+ (B cells) as well as CD94+ (NK cells and a subset of CD8+ T-cells) and slight increase of CD14+ cells (monocytes, macrophages) compared to PBS treated Balb/c mice (Figure 3A).

A cell suspension prepared from solid tumors of the studied mice showed presence of CD4+, CD8+, CD19+, CD94+ as well as CD14+ cell populations in the tumor microenvironment (Figure 3B). These results suggest a movement of immune cells populations to the developed tumors and a relevant decrease of the same populations in the spleen of the animals.

### Cytokine detection

TNFα, IFNγ, IL10 and IL4 levels were measured in mice sera using Enzyme linked immunosorbent assay (ELISA) kits. Challenge with 5×10^5^ C-26 carcinoma cells and treatment with RtH or HpH did not result in a significant difference in the cytokine production between the groups (data not shown).

### Detection of anti- C-26 antibodies formation

Groups of Balb/c mice were treated with Hcs from gastropods *R. thomasiana* and *H. pomatia*. Two groups (sensRtH, sensHpH) were injected with RtH or HpH 2 weeks before tumor cells inoculation and after establishment of palpable solid tumors the mice were injected intratumoraly once weekly with RtH or HpH.

The other two groups (RtH, HpH) were inoculated with 5×10^5^ cells/mouse of C-26 cells and after palpable solid tumor development the animals were injected once a week with RtH or HpH. Mice injected with C-26 cells only or PBS injected healthy mice were used as controls.

Collected sera from all studied groups were tested for presence of anti-C-26 antibodies.

**Figure 4:**
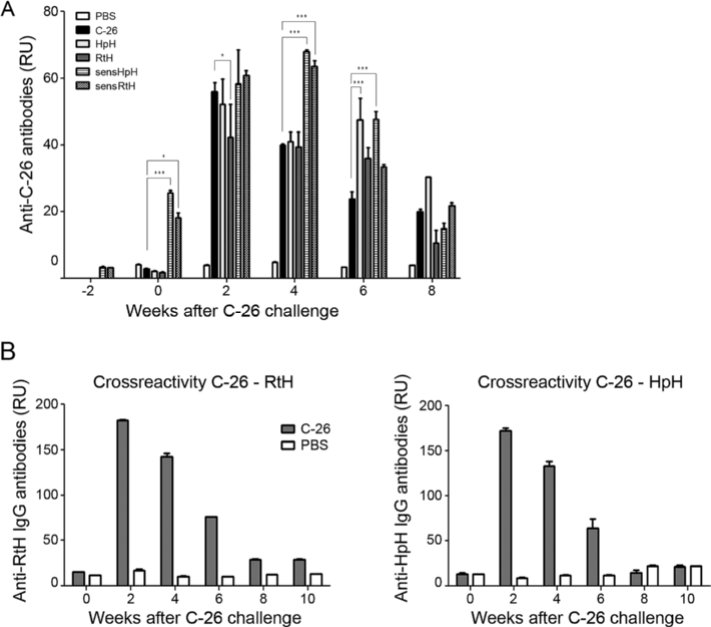
**Detection of anti- C-26 antibodies and anti-C-26 antibody cross-reactivity. (A)** Anti- C-26 antibody levels in C-26 bearing-mice treated with RtH or HpH (pretreated before C-26 inoculation with RtH, or HpH, or not treated) were measured by ELISA. Mice inoculated with C-26 cells without Hcs or mice treated only with PBS were used as control groups. The results are presented as Relative Units (RU) calculated according to the titer of standard anti-C-26 antibodies used for ELISA. The data are presented as mean ± SD. p values are calculated using the Two-way ANOVA test (*p < 0.05; ***p < 0.001), in comparison to C-26 bearing mice. **(B)** ELISA assays for anti-C-26 antibody cross-reactivity. 96-well ELISA plates were coated with HpH or RtH (20 μg /ml) and after blocking the plates were incubated with diluted sera from mice inoculated with C-26 colon carcinoma cells only or PBS healthy controls. The results are presented as Relative Units (RU). Mean ± SD values were calculated for each group.

All groups developed anti-C-26 IgG antibodies compared to the PBS treated control group (Figure 4A). The titer of these antibodies decreased in C-26 group without hemocyanin treatment during tumor development.

The level of anti-C-26 IgG antibodies in the groups immunized with RtH and HpH without priming did not differ compared to the level of C-26 control group for the first 4 weeks after cell administration. Further treatment with RtH or HpH prolonged persistence of high levels of anti-C-26 antibodies in the sera of these animals. A number of studies demonstrate the prolonged survival in case of enhanced anti-tumor antibody production [24].

The administration of RtH or HpH to sensRtH and sensHpH groups resulted in a presence of anti-C-26 antibodies even before cell inoculation which higher values were measured at the 4^th^ week after C-26 injection (Figure 4A). To determine whether the recognition of C-26 by antibodies generated after priming with Hcs is due to polyspecificity we tested the sera specifisity to foreign antigene (tetanus toxoid). We observed similar pattern of anibody titers to the pattern of anti-C-26 in all of the groups injected with Hcs (data not shown).

To examine the presence of cross-reactive epitopes in Hc molecules and C-26 cells we set ELISA with the HpH and RtH coated on the 96-well plates and incubated with sera from animals challenged with C-26 cells only. The results showed high levels of crossreactive IgG antibodies to HpH and RtH in the sera of animals after tumor cells inoculation (Figure 4B). These results suggest the presence of crossreactive epitopes within the hemocyanins and C-26 colon carcinoma cells. The biochemical nature of these epitopes needs to be studied and determined [25].

### Survival and tumor manifestation

**Figure 5:**
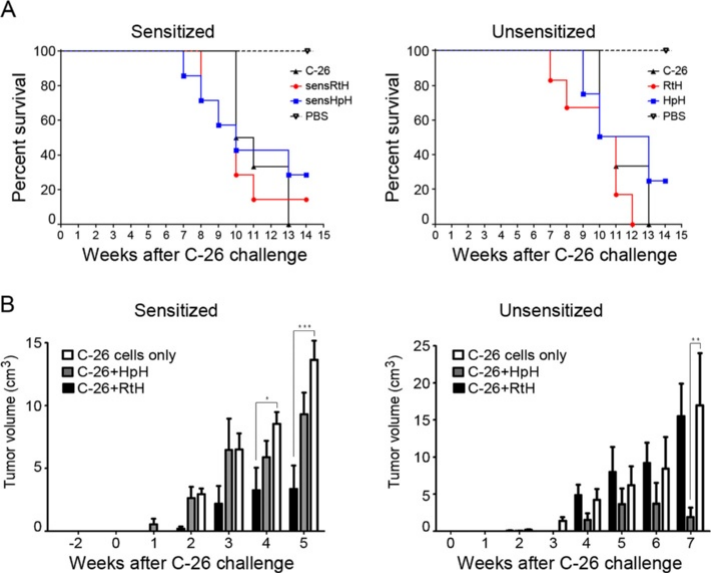
**Tumor weight and survival. (A)** Survival curves of studied animal groups. **(B)** The dynamics of tumor growth was monitored in all Hcs treated groups and compared to control group (C-26 only). The data are presented as mean ± SD. The p-values are calculated using the Two-way ANOVA test (*p < 0.05; **p < 0.01; ***p < 0.001), in comparison to C-26 bearing mice.

The survival of all tested groups was compared with the survival of animals inoculated with C-26 cells only. The results showed 15% survival of sensRtH group on week 15, 30% survival of the sensHpH group and over 30% survival of the group treated with HpH compared to the C-26 group while the group treated with RtH did not show improved survival (Figure 5A).

The dynamics of tumor growth was monitored in all Hcs treated groups and compared to the control group (C-26 only). Animals injected with RtH and HpH with or without priming developed small size tumors within the whole period of observation (Figure 5B). HpH treated group with no priming even showed a decrease in the tumor growth at week 7^th^ after C-26 challenge.

### Histology and organ comparison

**Figure 6:**
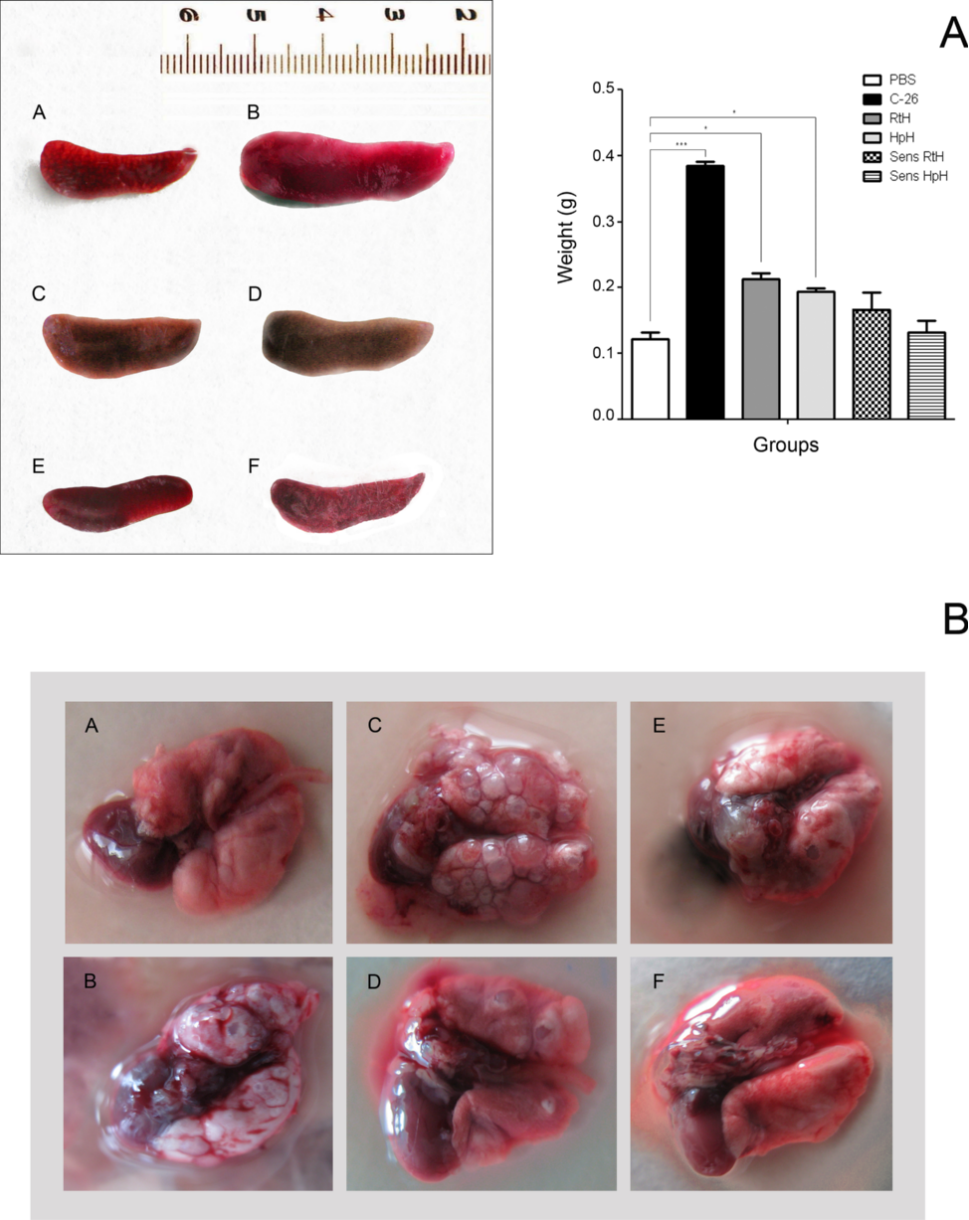
**Organ comparison. (A)**: Left panel: Spleen size comparison of PBS healthy control (A), C-26 bearing mice (B), C-26 bearing RtH treated animals (C), C-26 bearing HpH treated animals (D), sens RtH mice (E), sens HpH mice (F). Right panel: the weights of the spleens from Hcs treated animals compared to untreated mice. The data are presented as mean ± SD. The p-values are calculated using the Two-way ANOVA test (*p < 0.05; ***p < 0.001). **(B)** Comparison of metastatic manifestations in the lungs of PBS healthy control (A); C-26 bearing mice (B), C-26 bearing RtH treated animals (C), C-26 bearing HpH treated animals (D), sens RtH mice (E), sens HpH mice (F). Data are representative of at least 3 independent experiments.

Splenomegaly is a characteristic feature of C-26 murine tumor model [26]. Representative numbers of mice from each group were sacrificed for organ comparison and examination. We observed a more than 3-fold significant increase of the spleens in non treated C-26-bearing mice compared to Hcs treated and mostly it was expressed in sensHpH and sensRtH groups (Figure 6A).

Lungs from all studied groups were excised and the levels of metastatic manifestation were compared to the C-26 group. The Figure 6B shows large metastatic nodules formed in the lungs of the control mice and also in the RtH-treated group compared to the markedly inhibited number of surface metastasis in the sensRtH, sensHpH and in HpH treated groups.

We have examined morphological and histological features of C-26 tumor model. The paraffin sections of solid tumors from all studied groups were stained with heamatoxilin-eosin histological staining technique and were analyzed by light microscope. The tumor necrosis indicates a poor prognosis for different cancers and is associated with the survival. We observed differences into the necrosis sections within the tumor mass of the studied animals at 6^th^ week of the treatment schedule. In the sensRtH and sensHpH groups the levels of necrosis were less than in the RtH, HpH groups and also in the group of animals bearing C-26 carcinoma cells only (data not shown). These results could be associated with the enhanced antibody titers that we have found in the sera of the hemocyanin-pretreated animals and with prolonged survival of these groups.

## Discussion

There is a great need for new natural products and compounds with anti-cancer effects. The nature-derived substances can be formally separated into two groups i) targeted on the development of the tumor itself such as inhibitors of key for tumor growth molecules and ii) molecules enhancing the ability of immune cells to distinguish the non-self cancer cells and to induce adequate and potent anti-cancer response. The majority of them have high specificities and limited adverse effects compared to common therapies. The results from the current study confirm these statements.

Molluscan Hcs influence the apoptotic pathways of different cancer cell lines but the exact mechanisms are not known [27,28]. McFadden et al. presumed that the tumor suppressive effect of KLH could be mediated by alterations in apoptotic pathways. They have demonstrated that KLH has effect by increased apoptosis of SEG-1 cell line (human Barrett’s esophageal adenocarcinoma) with intact p53 tumor suppressive gene. On the other hand the KLH shows no apoptotic effect on BIC-1 cells (Barrett’s esophageal adenocarcinoma) with mutated p53 gene. It is one possible mechanism for induced pro-apoptotic effect of Hcs on the colon carcinoma cell line C-26 but this has not been confirmed so far and further study needs to be done.

Many authors performed proliferation assay (Wst8 or MTT) with Hcs, but to study apoptotic effect of gastropodan Hcs on tumor cells by FACS is more precisely [27,28]. In our hands we observed an increased apoptosis of tumor cells after Hcs treatment *in vitro*, but always some cells remained unaffected. The extensive proliferation of these unaffected cells could mask the Hcs treatment results.

C-26 carcinoma cells challenged Balb/c mice had a decrease of CD4+, CD8+, CD19+ as well as CD94+ cells in the spleen compared to PBS treated ones. In contrast, we have observed a presence of tumor-infiltrating lymphocytes, which can explain the migration of these cell populations from the spleen.

The immunization with RtH or HpH did not result in a significant difference in the cytokine profiles (TNFα, IFNγ, IL10, IL4) between the groups.

The presence of anti-C-26 antibodies generated after priming with RtH or HpH even before C-26 cell challenge could be explained with production of polyspecific antibodies recognizing Hc molecules or with existence of crossreactive epitopes on the surfaces of C-26 carcinoma cells and Hcs. Such epitope was already detected on the SBC cells and KLH [25]. It was observed a crossreactivity of anti-Hc antibodies to the tetanus toxoid and C-26 cells. This result reveals a broad spectrum of polyspecificity of generated antibodies after Hc administration.

In the beginning of Hcs administration some of the treated mice exhibited worse survival rate than the untreated ones. Finally, in the end-point HpH treated groups had better performance and exhibited improved survival compared to the control group. This observation illustrates that improvement of several clinical parameters (anti-C26 Antibodies development, reduction of metastatic manifestation and spleen size) does not always prolong survival.

## Conclusion

The Hcs - treated animals had prolonged survival and inhibited tumor growth, splenomegaly and lung metastasis appearance. These huge proteins can induce a vigorous anti-tumor adaptive immune response. Taken together our findings demonstrate that the Hcs isolated from different arthropod and mollusc species could be used as pro-apoptotic agents able to improve humoral anti-cancer response and moderate the manifestation of C-26 carcinoma symptoms.

## Methods

### Mice

Female 10 weeks old Balb/c mice were obtained from Harlan Farm, Blackthorn, UK. The animals were kept under specific pathogen free (SPF) conditions and the manipulations were approved by the Committee on Animal Research and Ethics at the Bulgarian Food Safety Agency - Ministry of Agriculture and Food in accordance with the international regulations.

### Antibodies

FITC (Fluorescein isothiocyanate) -conjugated anti-mouse CD8, PE (Phycoerythrin) -conjugated anti-mouse CD94 and anti-mouse CD19 and APC (Allophycocyanin) –conjugated anti-mouse CD4 mAbs (eBioscience, Frankfurt, Germany) were used for FACS (Fluorescence-activated cell sorting) experiments. AP (Alkaline phosphatase) –conjugated anti-mouse IgG (Sigma-Aldrich, Taufkirchen, Germany) were used for ELISA.

### Cell line

Murine colon carcinoma cell line C-26 (CT26.WT (ATCC® CRL-2638™)) was cultured in complete RPMI (Roswell Park Memorial Institute medium) 1640 medium (Gibco, Gaithersburg, MD) containing 10% FCS (fetal calf serum), 4 mM L-glutamine, 50 μM 2-mercaptoethanol and antibiotics at 37°С/5% CO_2_. The confluent monolayer cells (90%) were trypsinized and used for animal administration and solid tumor establishment.

### C-26 cell lysate preparation

C-26 cell line was cultured in complete RPMI 1640 medium (see above) and 90% confluent cell monolayer was trypsinized and centrifuged (110 × g, 10 min, 4°C). The pellet was collected and was put under 8-fold cycles of freeze-thaw (from −80°C to 37°C). After centrifugation (110 × g, 2 min) the supernatant was kept and the total protein was determined spectrophotometrically at λ = 280 nm.

### Isolation and purification of hemocyanins

RtH was isolated from the hemolymph of marine gastropod *Rapana thomasiana* as described in [29]. HpH was isolated from the hemolymph of terrestrial snails *Helix pomatia* according to [30]. The HpH was solubilized in 100 mM sodium acetate buffer, pH 5.7, and the β-Hc isoform precipitated by dialysis of HpH against 10 mM sodium acetate buffer, pH 5.3, as described elsewhere [31]. Then β-HpH was solubilized in 100 mM sodium phosphate buffer, pH 6.5, at a concentration of 27 mg/ml (stock solution).

RtH and β-HpH were further purified by gel filtration chromatography on a Sepharose 4B column (90 × 2.4 cm), equilibrated and eluted with 50 mM PBS buffer, pH 7.2. The purity of isolated RtH and β-HpH was controlled by SDS- and native PAGE as described in [29,31], and by transmission electron microscopy [32]. The protein concentrations were determined spectrophotometrically using the specific absorption coefficient *A*_278_°^.1%^ = 1.36 mg^−1^ ml cm^−1^ (20°C) for RtH (27) and *A*_278_°^.1%^ = 1.416 mg^−1^ ml cm^−1^ (20°C) for β-HpH [33], respectively.

Hc solutions were passed once through a Detoxi-Gel column in order to remove endotoxin contaminations (Detoxi-Gel column, Thermo Fisher Scientific, Rockford). The level of the remaining endotoxin was determinated by Limilus Amebocyte Lysate coatest gel (LAL) (Chromogenix AB, Molndal, Sweden).

### Apoptotic assay

The C-26 cells were cultured in complete RPMI 1640 medium and 90% confluent cell monolayer was incubated in the presence of different concentrations of HpH and RtH (100 μg/ml, 12.6 μg/ml, 1.6 μg/ml and 0.1 μg/ml) for 24, 48 and 72 hours at 37°С/5% CO_2_. Then cells were collected, washed and stained with Annexin V-FITC apoptosis detection Kit I (BD Biosciences Pharmingen, Erenbodegem, Belguim), containing Propidium iodide as DNA-binding dye according manufacturer’s instruction. The apoptosis of gated cells was analyzed within 15 minutes using flow cytometry (BD LSR II flow cytometer). The early (AnnV-FITC+, PI-) and late (AnnV-FITC+, PI+) apoptosis, as well as necrosis (AnnV-FITC-, PI+) were measured at different intervals (24, 48 and 72 h of incubation).

### Tumor model establishment

Four groups of Balb/c mice (10 animals each) were challenged s.c. in the right flanks with different number of C-26 murine carcinoma cells ( 1×10^4^, 1×10^5^, 5×10^5^, 5×10^6^ cells/mice). The animals were observed for 14 weeks.

### Treatment schedule

Two groups of female Balb/c mice (20 animals per group) were injected with RtH (sensRtH) or HpH (sensHpH) (200 μg/mouse, i.p.) 2 weeks before tumor cells inoculation (5×10^5^ cells/mouse). After palpable solid tumor establishment the animals were injected intratumoraly once weekly with 100 μg/mouse of RtH or HpH, respectively. Another two groups were inoculated with C-26 cells (5×10^5^ cells/mouse) and after palpable solid tumor development the mice were injected every week with RtH (RtH group) or with HpH (HpH group) (100 μg/mouse, intratumoraly). Control groups of mice were challenged with C-26 cells or PBS only. Another two control groups were injected once weekly with RtH or HpH only (100 μg/mouse, i.p.). Tumor growth was monitored by measuring palpable solid tumors once a week with a microcaliper and the tumor volume was determined. Every two weeks the animals from all groups were bled and the sera were kept frozen at −70°C. All experiments were duplicated.

### Flow cytometry analysis for phenotyping of spleen and tumor suspensions

Spleens and solid tumors from representative number of animals (n = 4 to 6) were taken and monocellular suspension was isolated by cell strainers (BD Biosciences, Erenbodegem, Belguim). The cells were washed with PBS (containing 2.5% FCS and 0.05% sodium azide) and incubated with one of the following anti-mouse antibodies - anti-CD4-APC, anti-CD8-FITC, anti-CD19-PE, or anti-CD94-PE. Each incubation step was performed for 30 min at 4°C. Finally, the cells were washed twice and kept at 4°C. Ten thousands cells were analyzed from each sample with a BD LSR II flow cytometer using the Diva 6.1.1. software (BD Biosciences, San Jose, CA).

### Cytokine quantification

TNFα, IFNγ, IL10 and IL4 levels were measured in mouse sera using ELISA sets (BD OptEIA™, BD Biosciences Pharmingen, Erenbodegem, Belguim) according to the manufacturer’s instructions.

### ELISA assays

#### ELISA assays for anti-C-26 and anti-TT IgG antibodies

96-well plates (Nunc, Roskilde, Denmark) were coated with C-26 lysate (150 μg/ml) or with tetanus toxoid (TT) (20 μg/ml, from Bulbio, Sofia, Bulgaria) diluted in coating buffer (NaHCO_3_, pH 9,6) by incubation overnight at 4°C. The plates were washed with PBS/0.05% Tween 20 and blocked with 1%BSA in PBS for two hours at room temperature. After washing diluted serum samples from different groups of animals were added and incubated for 1 hour at room temperature. The plates were then washed, incubated for 1 hour at room temperature with alkaline phosphatase-labelled goat anti-mouse IgG and developed by alkaline phosphatase substrate pNPP (p-Nitrophenyl Phosphate). After washing the absorbance was measured at 405 nm. The obtained ELISA results were presented as relative units (RU), corresponding to the titer of anti-C-26 or anti-TT standard antibodies used for ELISA.

#### ELISA assays for anti-C-26 antibody cross-reactivity

Another 96-well plates were coated with HpH or RtH (20 μg/ml) in coating buffer overnight at 4°C. After blocking, the plates were incubated with diluted sera from mice inoculated with C-26 colon carcinoma cells only. Plates were then developed as described above.

### Histology and organ comparison

Solid tumors were resected from terminal animals. After fixation in phosphate-buffered formalin (10%) the tissues were embedded in paraffin and 7 μm sections were analyzed using a standard haematoxylin/eosin staining technique.

Spleens, kidneys, livers and lungs were taken from the same animals and were compared with organs from healthy mice for size differences 8 weeks after the tumor cell administration.

### Statistical analysis

The values in figures correspond to mean ± SD. The Two-way ANOVA test was used to determine differences between each two groups. A value of P < 0.05 was considered as statistically significant. Survival significance was determined via analysis of survival curves with Prism software from GraphPad (San Diego, CA).

## Abbreviations

Hc: Hemocyanin
KLH: Keyhole limpet hemocyanin
RtH: *Rapana thomasiana* hemocyanin
HpH: Helix pomatia hemocyanin
